# Evaluation and improvement of angular response for a commercial 2D detector array for patient‐specific QA

**DOI:** 10.1002/acm2.14106

**Published:** 2023-08-18

**Authors:** Sha Wang, Yuanshui Zheng

**Affiliations:** ^1^ Guangzhou Concord Cancer Center Sino‐Singapore Guangzhou Knowledge City Guangzhou City China

**Keywords:** angular response, composite dose verification, IMRT, MatriXX, quality assurance (QA), VMAT

## Abstract

**Purpose:**

MatriXX ionization chamber array has been widely used for the composite dose verification of IMRT/VMAT plans. However, in addition to its dose response dependence on gantry angle, there seems to be an offset between the beam axis and measured dose profile by MatriXX for oblique beam incidence at various gantry angles, leading to unnecessary quality assurance (QA) fails. In this study, we investigated the offset at various setup conditions and how to eliminate or decrease it to improve the accuracy of MatriXX for IMRT/VMAT plan verification with original gantry angles.

**Methods:**

We measured profiles for a narrow beam with MatriXX located at various depths in increments of 0.5 mm from the top to bottom of the sensitive volume of the array detectors and gantry angles from 0° to 360°. The optimal depth for QA measurement was determined at the depth where the measured profile had minimum offset.

**Results:**

The measured beam profile offset varies with incident gantry angle, increasing from vertical direction to lateral direction, and could be over 3 cm at vendor‐recommended depth for near lateral direction beams. The offset also varies with depth, and the minimum offset (almost 0 for most oblique beams) was found to be at a depth of ∼2.5 mm below the vendor suggested depth, which was chosen as the optimal depth for all QA measurements. Using the optimal depth we determined, QA results (3%/2 mm Gamma analysis) were largely improved with an average of 99.4% gamma passing rate (no fails for 95% criteria) for 10 IMRT and VMAT plans with original gantry angles compared to 94.1% using the vendor recommended depth.

**Conclusions:**

The improved accuracy and passing rate for QA measurement performed at the optimal depth with original gantry angles would lead to reduction in unnecessary repeated QA or plan changes due to QA system errors.

## 1 INTRODUCTION

Intensity‐modulated radiation therapy (IMRT) and volume modulated arc therapy (VMAT) are widely used in radiation therapy currently due to their improved dose conformity to the target volumes while potential reduction of dose to the surrounding healthy tissues. Due to the complexity of IMRT and VMAT, thorough verification of planned radiation dose before treatment is necessary to ensure that the treatment plan can be executed accurately.^1^, ^2^ 2D arrays with ionization chambers, such as the MatriXX QA system developed by IBA dosimetry (Schwarzenbruck, Germany), are widely used for dose verification of IMRT^3^, ^4^, ^5^, ^6^, ^7^, ^8^, ^9^, ^10^, ^11^ and VMAT.^12^, ^13^, ^14^, ^15^, ^16^, ^17^, ^18^, ^19^, ^20^, ^21^ Dobler et al.^15^ investigated the angular dependence of the 2D ionization chamber array I'mRT MatriXX for oblique beam incidence and its effects for plan verification of IMRT with original gantry angles. They suggested that a correction factor for oblique gantry angle is required to obtain good agreement between the calculated and measured dose distribution. Wolfsberger et al.^18^ further investigated the angular dependence of MatriXX and its correction method, assuming that the angular correction factor is constant within the detector plane for any given gantry angle. Boggula et al.^22^ studied the angular dependence of all detectors of MatriXX and used their angular correction for compound dose verification with VMAT. Shimohigashi et al.^23^ studied the angular dependence of central and off‐axis detectors and used these correction factors for compound dose validation of IMRT. However, during our IMRT/VMAT QA program commissioning, we found that the discrepancy between the calculated dose profile by treatment planning system (TPS) and measured by MatriXX^Evolution^ sometimes could still be high and lead to relatively too frequent QA fails during dose verification of IMRT/VMAT with original gantry angles even dose calibration with angular correction as mentioned in previous studies and operation procedures as recommended by vendor are closely followed.

We investigated the root cause of the discrepancy and found that there is offset between the beam central axis and measured dose profile by MatriXX for oblique beam incidence. To our knowledge, this phenomenon has not been reported yet. Therefore, the aim of this study was to investigate the profile offset for oblique beam incidence under various MatriXX setup conditions and how to account for or minimize its effect to further improve the accuracy of MatriXX for dose verification of VMAT and IMRT plans with original gantry angles.

## 2 MATERIALS AND METHODS

### 2.1 Linear accelerator and treatment planning systems

All the measurements were performed on a Truebeam linear accelerator (Varian Medical Systems, Palo Alto, CA) with 6 MV, 10 MV, and 6 MV FFF photon beams. Eclipse treatment planning system (v15.6, Varian Medical Systems, Palo Alto, CA) with anisotropic analytical algorithm (AAA) was used to generate IMRT and VMAT plans as well as the corresponding QA plans.

### 2.2 MatriXX^Evolution^ system

**FIGURE 1:**
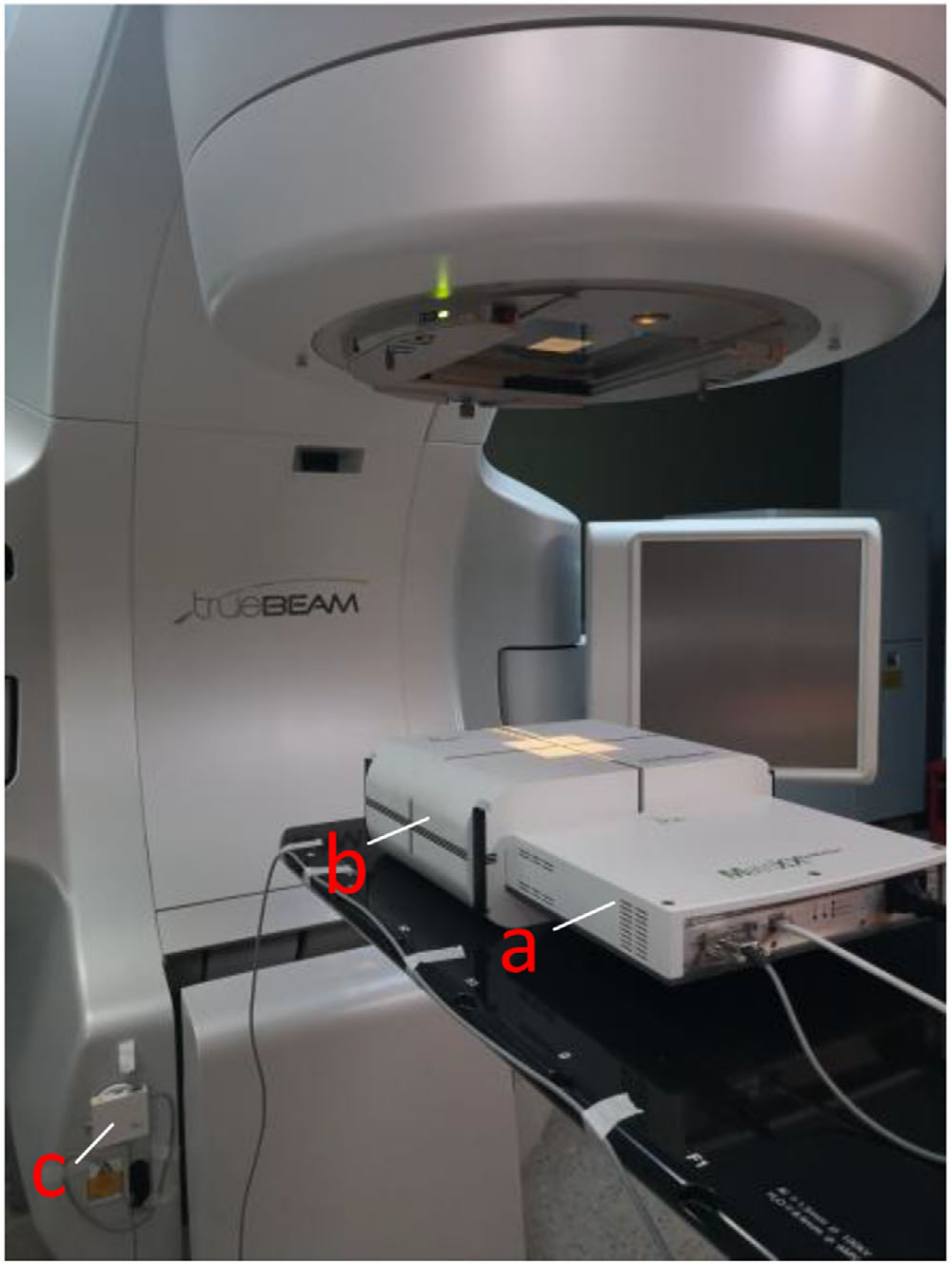
Measurement setup of the MatriXX patient QA system, which includes the MatriXX^Evolution^ device (a), inserted into the miniPhantom (b), and a gantry angle sensor (c).

The MatriXX^Evolution^ system is an upgraded version of the MatriXX QA series developed by IBA dosimetry for IMRT/VMAT QA with original gantry angles. It consists of a MatriXX 2D detector array device, a miniPhantom, a gantry angle sensor (GAS), and myQA software (v2.13, IBA Dosimetry). The design and construction of MatriXX were reported by Amerio et al.^4^ and described in the vendor manual.^24^ The MatriXX is made of an ionization chamber array with 1020 chambers arranged in a 32 × 32 grid (except for the four corner positions without chambers). The chamber array is of 7.62 mm for each neighboring chamber center‐to‐center and covers an active field of 24.4 cm × 24.4 cm at 100 cm source to detector distance (SDD). Each chamber is 4.5 mm in diameter and 5 mm in height. The miniPhantom is a solid phantom made of the RW3 white‐polystyrene material which is water equivalent for the radiation therapy treatment energy. The system includes a GAS, which is attached to the Linac gantry head to obtain the gantry angle when the MatriXX phantom is irradiated for dose measurements. Figure 1 shows the measurement setup of the MatriXX patient QA system. myQA software was used to analyze the measurement data and perform patient specific QA.

### 2.3 Output calibration for absolute dose and angle response correction

Before its use for QA measurements, the absolute dose response of MatriXX was calibrated using an ionization chamber FC‐65G (IBA Dosimetry) according to the manufacturer's instructions. MatriXX output calibration for absolute dose is compared with the FC‐65G chamber measurement under the same condition of SSD 100 cm, measurement depth 10 cm, field size 10 × 10 cm^2^, and 100 MU.

To compute the correction factor as a function of angle (θ), the true dose for each detector at each angle must be first determined. Here we assume the TPS calculated dose on the MatriXX QA phantom with the same setup and gantry angle as the true dose. Therefore, the correction factor *C_ij_(*θ) for a MatriXX pixel at row *i* and column *j* at gantry angle θ is defined as

(1) $$C_{ij}\left(\theta \right)\;=N_{ij}\;\frac{D_{ij}^{ref}\left(\theta \right)}{D_{ij}^{meas}\left(\theta \right)}$$

where $D_{ij}^{ref}\left(\theta \right)$ is the TPS calculated dose at the center of ionization chamber and $D_{ij}^{meas}\left(\theta \right)$ are the the MatriXX measured dose at gantry angle θ, chamber at row *i* and column *j*, and *N_ij_ (*θ*)*, a normalization factor that normalizes *C_ij_* (0°) = 1, that is, the ratio of measured dose to the reference dose for chamber *ij* at angle 0.

We measured the doses *D^meas^* with a 10° step for gantry angles (θ) of 0°−70° & 110°−180°, a 2° step for angles of 70°−80° & 100°−110°, and a 1° step for angles of 80°−100° in miniPhantom, with a 30 × 10 cm^2^ field for 6 MV, 6 MV FFF, and 10 MV photon beams. $D_{ij}^{meas}\left(\theta \right)$ was measured at the vendor‐recommended depth initially and repeated at the optimal depth we determined later on. 30 × 10 cm^2^ field was used to fully cover all columns of the ionization chamber array while protecting the electronic component of the detector per vendor recommendation.

### 2.4 Measurements of beam profile offset

**FIGURE 2:**
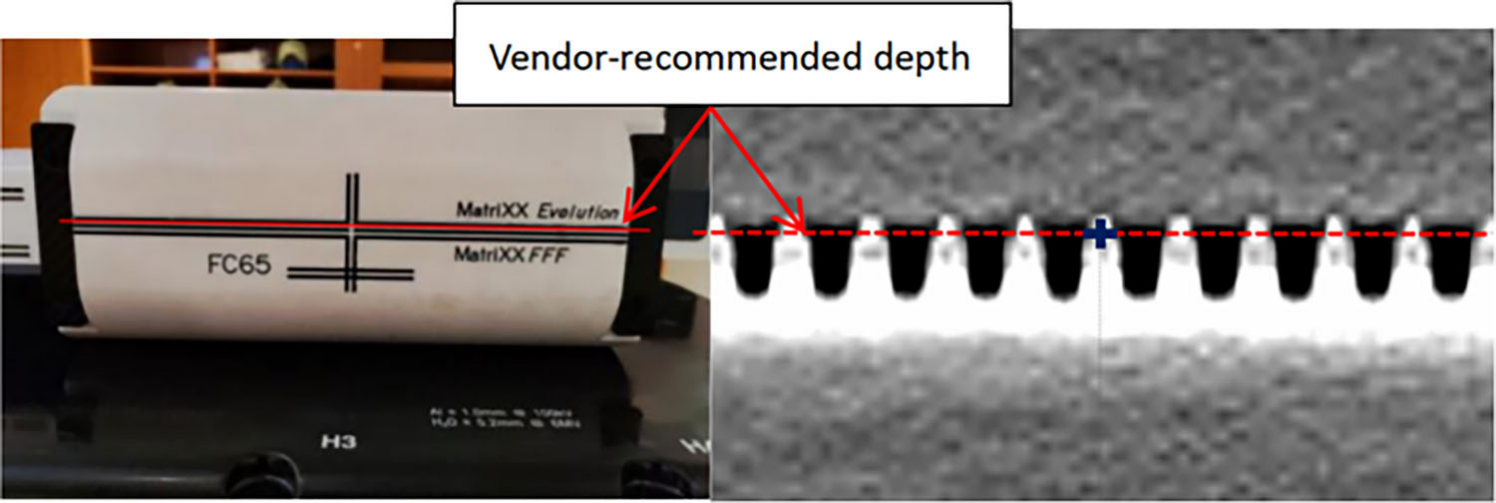
Illustration of correlation of the alignment markers on the miniPhantom (left) and the vendor‐recommended depth aligned to isocenter on the scanned CT images in the TPS (right).

**FIGURE 3:**
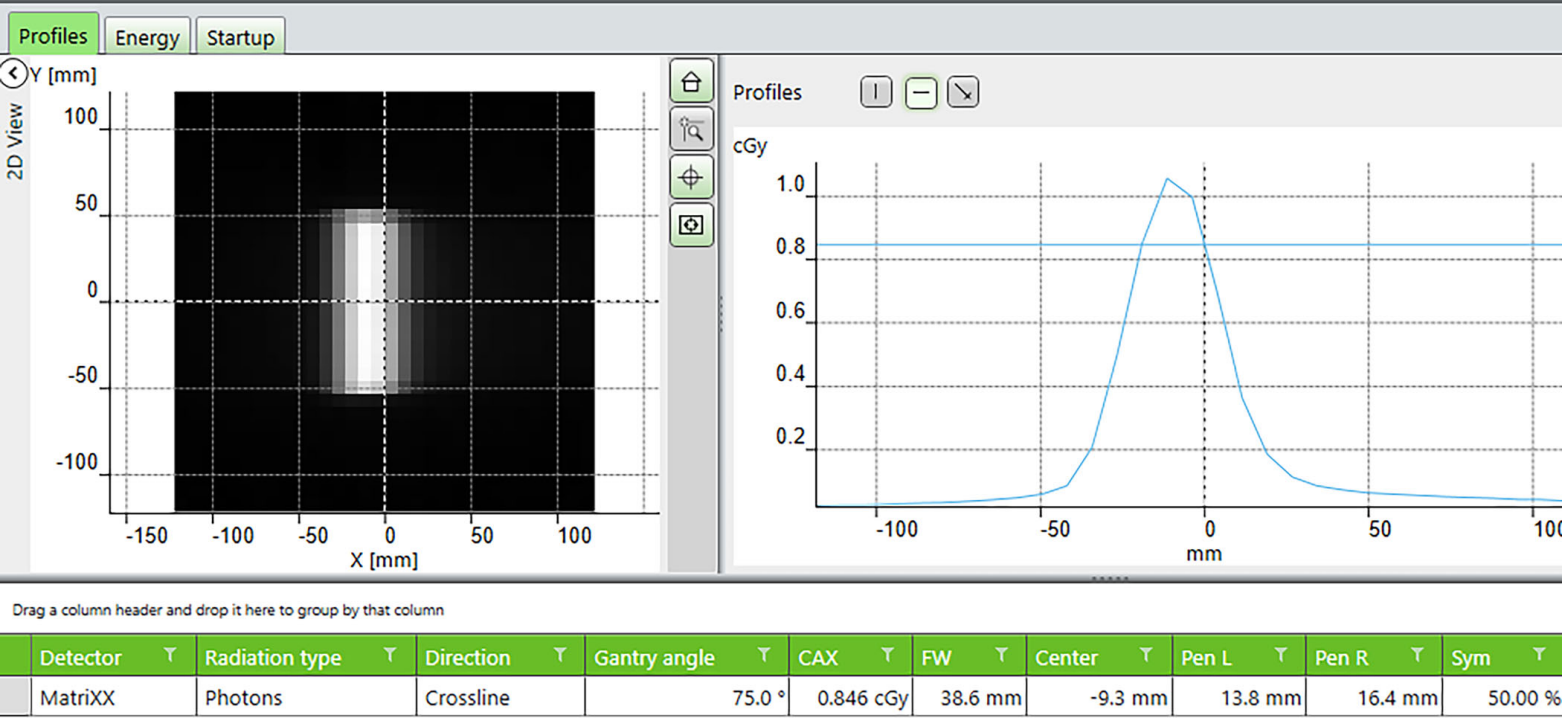
The beam profile offset measured by MatriXX for a narrow beam at gantry angle 75° using the application module “FastTrack” of the myQA software.

We first measured the offset as a function of gantry angle at the vendor‐recommended depth, where the “MatriXX^Evolution^”marker line on the miniPhantom was aligned to the isocenter, as indicated in Figure 2. The Miniphantom with MatriXX^Evolution^ inside was placed flat on the treatment couch. MatriXX^Evolution^ center was aligned to the beam center when both gantry and collimator were set to 0°. A narrow beam of 0.5 cm in lateral (X) and 10 cm in longitudinal (Y) was irradiated to the MatriXX for each gantry between 0 and 180° in increments of 5°, and repeated between 180° and 360° in increments of 10° except 270° was replaced by two angles of 265° and 275°. The use of this narrow field allowed to obtain a full width profile at all gantry angles for profile offset calculation. The beam profile was analyzed and the center of the profile measured by the MatriXX from beam axis was recorded as the offset using the application module “FastTrack” of the myQA software, as shown in Figure 3.

To study the effect of the depth placement for MatriXX on beam profile offset, the MatriXX was moved up and down so that the isocenter was aligned to different positions from top to bottom of the sensitive volume of the array detectors in increments of 0.5 mm. For each position, the profile was measured for gantry angles of 0, 180° and from 30° to 150° with increments of 15°. The offset of MatriXX measurement as a function of measurement depth was analyzed and an optimal depth was determined at which the minimum offset by the MatriXX was found. At the optimal depth, we re‐calibrated the MatriXX for absolute dose and angle response for clinical patient QA measurements.

To verify the optimal depth for various beam energies and phantoms, we repeated the beam offset for other energies, 10 MV and 6 MV FFF in addition to 6 MV, at various gantry angles from 0 to 180°. In addition, to verify the optimal depth for various phantoms, we added 2 cm and then 5 cm solid water phantom to the miniPhantom and repeated the measurement of beam profile offset for gantry angle 45° at both the vendor‐recommended depth and the optimal depth.

### 2.5 Dose verification of patient‐specific IMRT and VMAT plans at optimal depth

IMRT and VMAT test plans with original gantry angles for different patients were created for the composite dose verification. In total 10 plans were created for 7 test patients with below cancer sites: brain, esophagus, liver, cervical, nasopharynx, lung, and prostate cancer. The 10 plans include a combination of various energies (6 MV, 6 MV FFF, and 10 MV) and treatment techniques (IMRT and VMAT), as listed in Table 1.

**TABLE 1:** The details for 10 IMRT and VMAT plans for different patients.

Plan no.	Site	Energy	Technology	Gantry angle
1	Brain	6 MV	IMRT	0°, 36°, 72°, 108°, 324°
2	Esophagus	6 MV	IMRT	120°, 150°, 180°, 210°, 240°
3	Liver	6 MV	IMRT	15°, 160°, 290°, 310°, 355°
4	Cervical	6 MV	IMRT	0°, 51°, 103°, 154°, 206°, 257°, 309°
5	Nasopharynx	6 MV	IMRT	9 beams from 0 to 320° at 40° interval
6	Lung	6 MV FFF	IMRT	15°, 40°, 70°, 120°, 150°
7	Cervical	10 MV	IMRT	0°, 51°, 103°, 154°, 206°, 257°, 309°
8	Prostate	6 MV	VMAT	1 full arc (181° to 179°)
9	Cervical	6 MV	VMAT	2 full arcs (181° to 179°, 179° to 181°)
10	Nasopharynx	6 MV	VMAT	2 full arcs (181° to 179°, 179° to 181°)

For each plan, a QA verification plan was created by applying the same plan to the MatriXX QA phantom, which was scanned with a GE RT Discovery CT Scanner (GE Medical System, United States) with 1.25 mm slice thickness. A treatment couch was added to the phantom to account for its attenuation for beams treating through the couch. For each patient plan, two QA plans were generated in TPS and measured with the MatriXX, one with the isocenter aligned to the vendor‐recommended depth and the other to the optimal depth determined in our study. The calculated dose distribution for each QA was exported to the application module “myQA Patients” of myQA software. Gamma analysis was used to compare and analyze the dose distributions of MatriXX and TPS. The gamma index was calculated using a 3% dose difference and 2 mm distance to agreement (DTA) criteria with a 10% threshold to exclude the low‐dose region as recommended by AAPM report TG218.^25^ For QA plans failed at the vendor‐recommended depth, we performed beam‐by‐beam analysis and beam profile comparison to investigate the cause of QA failure.

## 3 RESULTS

### 3.1 Angular gain response correction

**FIGURE 4:**
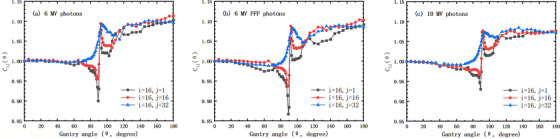
Angular response correction for IC units at the left (*j* = 1), central (*j* = 16), and right (*j* = 32) columns and the central row (*i* = 16) for the Matrix detector array for photon energies of 6 MV (a), 6 MV FFF (b), and 10 MV (c).

The measured dose gain response as a function of gantry angle was measured for each unit in the 2D ionization chamber array of the MatriXX system. Figure 4 shows the angular response correction for units at various columns at the central (16th) row. The correction factor was normalized at gantry 0, and ranged from around 0.87 to 1.12. The angular response is slightly different among energies, but is similar in shape for all energies.

### 3.2 Beam profile offset at vendor‐recommended depth

**FIGURE 5:**
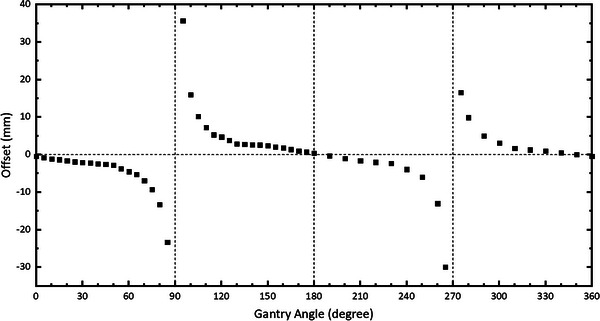
The profile offset as a function of gantry angle measured at vendor‐recommended depth.

The beam profile offset for a narrow beam was measured at various gantry angles, as shown in Figure 5. There is no offset at gantry 0, and the amount of offset gradually increases with the gantry angle, within 3 mm when gantry angle is less than 50°. The offset then increases more quickly when it is close to 90°, for example, about 23 mm at 85°. The same trend follows when the gantry angle varies from 180° to 90°, with the offset in the opposite direction for angles between 0 and 90°. The offset at various gantry angles from 180° to 360° is similar to that from 0° to 180° in both direction and magnitude, with some noticeable difference mainly at large oblique angles (close to horizontal beam).

### 3.3 Beam profile offset at different depths

**FIGURE 6:**
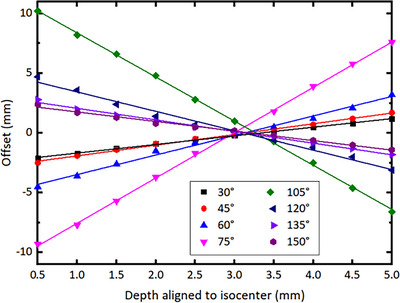
The beam profile offset measured by MatriXX as a function of measurement depth aligned to isocenter for various incident gantry angles. Lines are linear fitting of data for each gantry angle.

Table 2 shows the beam profile offset measured by MatriXX with isocenter aligned at various depths and gantry angles. From Table 2, it can be seen that the variation of the profile offset with gantry angle at a certain depth is consistent with the results at vendor‐recommended depth as described previously. For the same incident angle, the profile offset varies linearly with the depth, as shown in Figure 6. At depths from the upper surface to the lower surface of the ionization chamber, the offset first decreases and then increases, with the minimum offset at depth of 3 mm from the upper surface of the chamber air cavity, that is, 2.5 mm below the recommended measurement depth marked by the manufacturer. As measurements at this depth have almost zero offset for all incident gantry angles, we referred it as the optimal depth for MatriXX QA measurements.

**TABLE 2:** The profile offset at different depths and different gantry angles. Depth *d* was defined from the upper surface of the ionization chamber air cavity.

	Depth aligned to isocenter *d* (mm)									
θ	0.5	1.0	1.5	2.0	2.5	3.0	3.5	4.0	4.5	5.0
30°	−2.1	−1.7	−1.3	−0.9	−0.5	−0.2	0.1	0.5	0.8	1.2
45°	−2.5	−1.9	−1.4	−0.9	−0.5	−0.1	0.3	0.7	1.2	1.7
60°	−4.5	−3.6	−2.6	−1.5	−0.8	−0.1	0.5	1.2	2.1	3.2
75°	−9.3	−7.7	−5.7	−3.7	−1.7	−0.1	1.8	3.9	5.8	7.6
105°	10.2	8.2	6.6	4.8	2.8	1.0	−0.7	−2.5	−4.6	−6.6
120°	4.7	3.6	2.4	1.4	0.7	0.1	−0.6	−1.2	−2.0	−3.1
135°	2.8	2.0	1.5	1.0	0.5	0.1	−0.3	−0.8	−1.3	−1.8
150°	2.4	1.7	1.3	0.8	0.5	0.2	−0.2	−0.6	−1.0	−1.4

### 3.4 Verification of the optimal depth for various photon energies and phantoms

Using the optimal depth we determined for 6 MV beams, we measured the beam offset for other energies, 10 MV and 6 MV FFF, at various gantry angle from 0° to 180° to verify whether the optimal depth is energy dependent. As shown in Table 3, the beam profile offsets measured by MatriXX for 10 MV and 6 MV FFF photon beams are also minimum, close to 0 except for large oblique gantry angles, where measurement uncertainty could be relatively high. Therefore, the optimal depth for MatriXX^Evolution^ seems energy independent and can be used for other energy treatment plan QA.

**TABLE 3:** The beam profile offset measured by MatriXX at the optimal depth for 6 MV FFF and 10 MV photons for various gantry angles.

6 MV FFF				10 MV			
Angle (°)	Offset (mm)	Angle (°)	Offset (mm)	Angle (°)	Offset (mm)	Angle (°)	Offset (mm)
0	0	180	0.2	0	0	180	0
30	0.2	150	0.2	30	0.2	150	−0.1
45	0.2	135	0.2	45	0.2	135	−0.1
60	−0.2	120	0.3	60	0.2	120	−0.1
75	−1.0	105	1.6	75	0.3	105	0.8
85	−1.2	100	3.5	80	0.8	100	2.5

Compared to the miniPhantom alone, no change was observed within the measurement uncertainty for the beam profile offset when 2 or 5 cm was added to the miniPhantom at vendor‐recommended depth and the optimal depth, indicating that the optimal depth for the Matrixx chamber array was independent of the phantom system, with which it may be used together.

### 3.5 Dose verification of VMAT and IMRT plans

Plan QA measurement for the 10 test plans were performed at the optimal depth we determined and compared with the result at the depth recommended by vendor. As shown in Table 4. The gamma evaluation results (3%/2 mm criteria) at the optimal depth are much improved compared to the vendor‐recommended depth, average of 99.4% versus 94.1%, and consequentially the number of patients passing the QA was also largely improved (10 vs. 4 for a 95% passing criteria).

**TABLE 4:** Comparison of gamma index values γ (3% dose and 2 mm DTA tolerance) for 10 IMRT and VMAT plans between the optimal depth and vendor‐recommended depth. All 10 plans measured at the optimal depth passed the QA (95% criteria) while 6 (highlighted in bold) failed when measured at the depth marked by manufacturer.

Plan no.	γ at vendor‐ recommended depth	γ at optimal depth
1	**94.6%**	100.0%
2	98.3%	100.0%
3	**92.4%**	100.0%
4	95.3%	100.0%
5	**92.3%**	99.2%
6	**89.8%**	98.7%
7	**91.0%**	99.6%
8	95.5%	97.6%
9	97.4%	99.8%
10	**94.7%**	100.0%
Average	94.1%	99.4%

**FIGURE 7:**
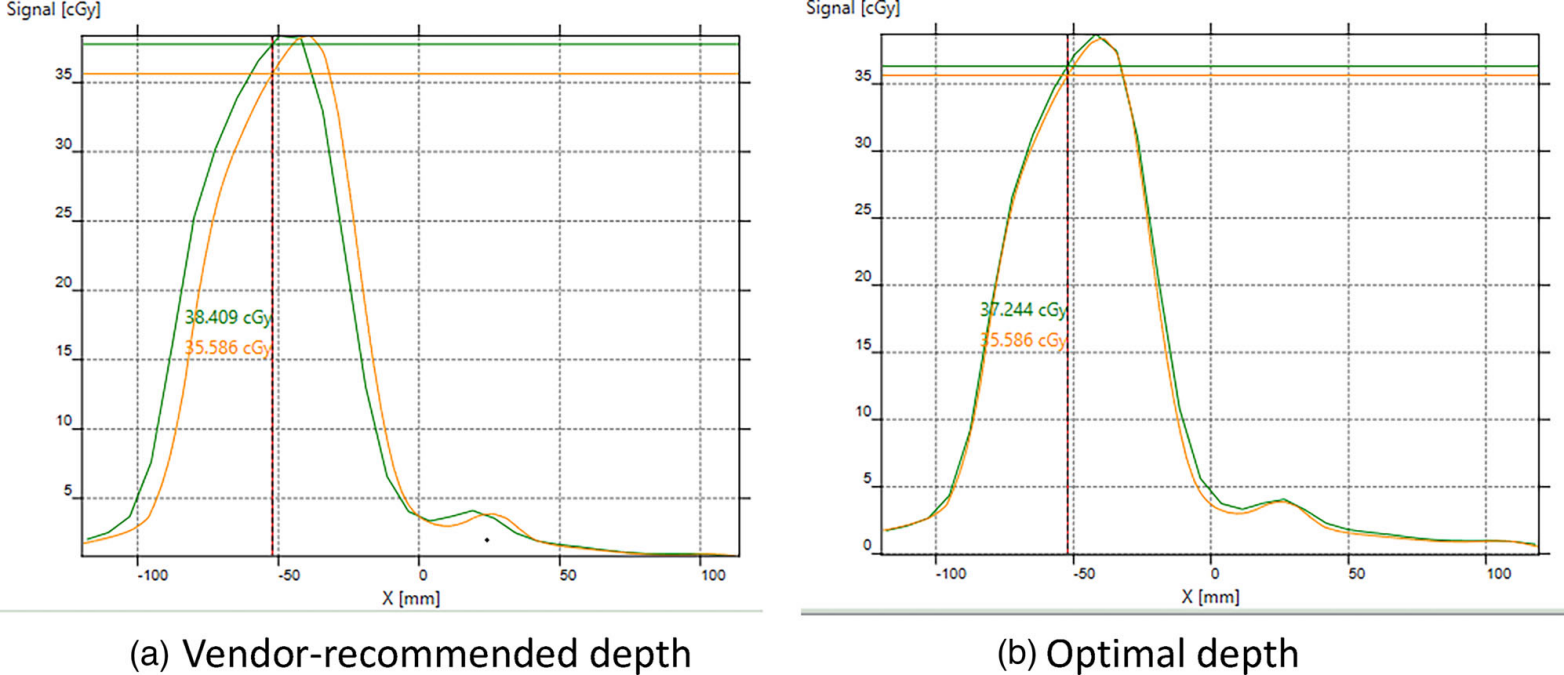
Comparison of beam profile for a gantry angle 240° field of an IMRT plan for a nasopharyngeal cancer case between the TPS calculation (orange) and the MatriXX measurement (green) at the vendor‐recommended depth (a) and the optimal depth (b). Angular gain correction was applied for Matrixx measurement at both depths.

For IMRT cases that failed at the vendor‐recommended depth, we performed beam‐by‐beam analysis in addition to the composite dose analysis. For large oblique beam angles, an offset was clearly seen when measured at the vendor recommended depth, but disappeared at the optimal depth, an example of which is shown in Figure 7. The elimination or reduction of beam profile offset at the optimal depth let to a large improvement of Gamma analysis passing rate.

## 4 DISCUSSION

In this study, we found that the angular response of the MatriXX system not only depended on the incident gantry angle, but also depended on the depth at which the MatriXX was aligned to the isocenter. Our data on the detector gain dependence as a function of gantry angle shows a similar pattern to previous studies.,^15^, ^18^, ^22^, ^23^ although the exact amount of correction factor differs slightly. It is notable that our gain correction factor was calculated according to the definition from the manufacturer, that is, the ratio of calculation to measurement, which was actually the reciprocal of what calculated by the formula (ratio of measurement to calculation) from Shimohigashi et al.^23^

To our knowledge, the dose profile offset with gantry angle with an ionization chamber detector array has not been reported yet. We studied the offset as a function of gantry angle and the detector depth aligned to isocenter, and found that when the MatriXX was aligned to isocenter plane at certain depth (the optimal depth), the beam dose profile offset would be eliminated or minimized for oblique gantry angles, thus largely improving the MartiXX response accuracy for IMRT/VMAT plan QA using original gantry angles. As shown in Figure 6, for very large oblique gantry angles, such as angles between 75° and 105°, there might still be non‐negligent beam profile offset even when the MatriXX is aligned with the isocenter at the optimal depth (3 mm from the ionization chamber surface as we determined), leading to potential QA failure if large oblique angle beams are used in an IMRT plan.

**FIGURE 8:**
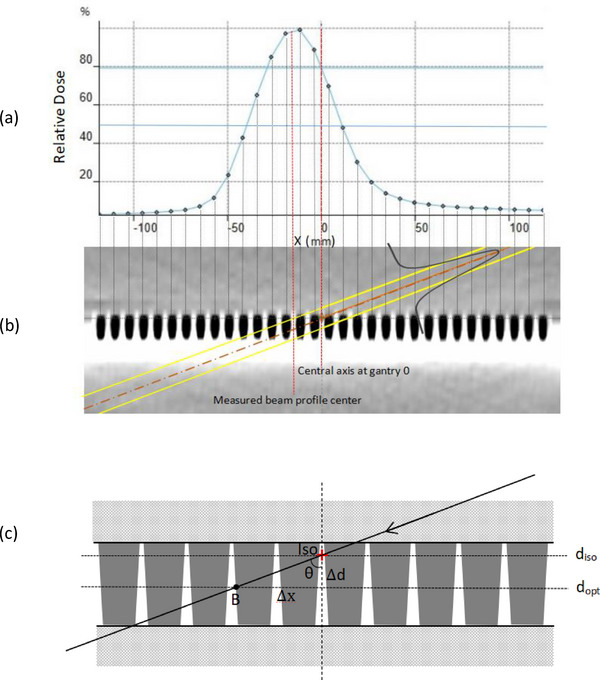
The schematic diagram to illustrate the beam center offset at oblique gantry angles. When a narrow beam at an oblique angle *θ* passes through isocenter not aligned to the optimal depth of measurement for the MatriXX, its measured beam profile center will be at point B, which is Δ*x* from the central axis at gantry 0. The magnitude and direction of offset depends on gantry angle *θ* and the distance (Δ*d*) between isocenter depth *d_iso_* and optimal depth *d_opt_*. When the isocenter is aligned to the optimal depth, there will be no offset for beams of all angles.

The beam profile offset for oblique beams at non‐optimal depth could be explained as follows. Since radiation beam may come from all directions instead of being perpendicular to the surface of the detector array, it does not hold anymore that the effective point of measurement is at the top surface of a parallel chamber, as typically used for percent depth dose measurement. When an oblique narrow beam passes through the detector array, it will deposit energy and release charge along its beam path in the sensitive volume of all chambers it passes through. The charge will be collected by these chambers and converted to dose signal for corresponding voxels the chambers represent. Therefore, the center of the beam profile for a narrow beam will be approximately corresponding to the center of the sensitive volume in all chambers that it passes through, as indicated in Figure 8a,b. As a result, the beam profile center will be offsetted by Δ*x* from the isocenter (Figure 6c). The offset Δ*x* will increase with the depth away from the central plane of sensitive volume (i.e., the optimal depth), and the gantry angle away from vertical direction, following a geometric triangle relation and can be calculated by

(2) Δx=Δd·tan(θ).

where Δ*d* is the distance between isocenter depth *d_iso_* and optimal depth *d_opt_*, and *θ is* the gantry angle.

When the isocenter is aligned to the optimal depth, there will be no offset for beams of all angles.

**FIGURE 9:**
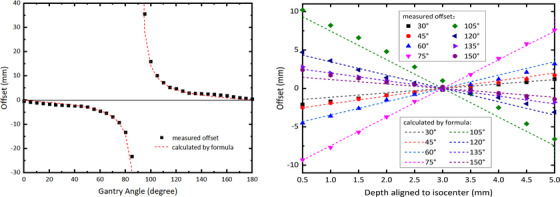
Comparison of beam profile offset calculated by our formula (Equation 2) with Matrixx measurement as a function of gantry angle as shown in Figure 4 (left), and at various depths as listed in Table 1 (right).

Using Equation (2), we calculated the beam profile offset as a function of gantry angles when the MatriXX was placed at the vendor‐recommended depth, as well as a function of depth for various incident gantry angles, and compared them to our measurements. As shown in Figure 9, the calculated offset was in excellent agreement with the measured result, within 1 mm except for beams with very large oblique angles. The reason for relative large difference for large oblique gantry angles could be caused by complicated geometry of the sensitive volume and wall structure of the chamber array and the resulting interactions between the beam and the multiple chambers it passes through.

Our study shows that the effective point of measurement, typically determined to be at the top surface of a parallel chamber in‐depth dose measurements, is not suitable for plan QA measurements with oblique gantry angles. Instead, an optimal depth for all gantry angles is roughly at the center but a little lower of the sensitive volume. The reason it is not at the exact geometric center might be due to the not fully symmetrical shape of chamber cavity and wall as well as their complex interaction with the beam. Therefore, the optimal depth is best determined by measurements, and should be used for QA measurements. When the measurement depth is not at the optimal measurement depth, the measured beam profile could be offsetted over 3 cm for large oblique incidence angles, leading to unnecessary QA failures due to systematic error in QA equipment, rather than the inaccuracy of the TPS calculation or limitation of the treatment delivery system.

## 5 CONCLUSIONS

MatriXX QA could fail due to beam profile center offset for oblique beams when it was aligned to a nonoptimal depth during QA measurements. Using the optimal depth determined in this study, the beam profile offset can be eliminated or minimized for oblique beams, thus improving the accuracy of QA measurement with original gantry angles for VMAT/IMRT plans, and avoiding potential unnecessary repeated QA or plan changes due to QA system errors.

## AUTHOR CONTRIBUTIONS

Sha Wang: Measurements, data analysis, and writingoriginal draft (lead). Yuanshui Zheng: Conceptualization, project administration, writing‐original draft (support), writing‐review, and editing.

## CONFLICT OF INTEREST STATEMENT

The authors declare no conflicts of interest.
